# Editorial: Exploration of Natural Product Leads for Multitarget-Based Treatment of Cancer—Computational to Experimental Journey

**DOI:** 10.3389/fphar.2022.850151

**Published:** 2022-02-22

**Authors:** Rajeev K. Singla, Marcus T. Scotti, Supratik Kar

**Affiliations:** ^1^ Institutes for Systems Genetics, Frontiers Science Center for Disease-Related Molecular Network, West China Hospital, Sichuan University, Chengdu, China; ^2^ iGlobal Research and Publishing Foundation, New Delhi, India; ^3^ Postgraduate Program in Natural and Synthetic Bioactive Products, Federal University of Paraíba, João Pessoa, Brazil; ^4^ Interdisciplinary Center for Nanotoxicity, Department of Chemistry, Physics and Atmospheric Sciences, Jackson State University, Jackson, MS, United States

**Keywords:** secondary metabolites, natural anti-cancer agents, herbal medicine, anti-cancer lead, chembioinformatics

Natural products are being used for the treatment of various diseases and disorders. A series of studies are undergoing from computational to clinical level to assess the translational potential of natural products (Scotti et al., 2016; Singla and Dubey, 2019; Singla et al., 2019; Kapusta et al., 2020; Singla et al., 2021a; Bansal et al., 2021; Singla et al.; Singla et al., 2021c; Dk and Nj, 2021; Singla et al.; Singla et al., 2021e; Singla et al.; Madaan et al., 2021; Marzocco et al., 2021; Okoh et al.; Talukdar et al., 2021). Cancer is one of the major public health burdens significantly impacting on a global level to both developed as well as the developing countries (Bray et al., 2018; Singla et al., 2021e; Qi et al., 2021; Joon et al., 2022). Specifically for the poor and underdeveloped countries, it is quite important to find cost-effective and sustainable resources for the treatment and management of cancer, as it will reduce the financial strain on them (Suryanarayana Raju et al., 2015; Singla et al.). Further, the high vulnerability to the point mutations leading to the development of resistance to the existing anticancer drugs, it is relevant to explore new multitarget leads to overcome resistance. This research topic was thus devised to collect the studies where natural products have been investigated for their multitarget potential against cancer.

Liu et al., in their article “Targets and Mechanism Used by Cinnamaldehyde, the Main Active Ingredient in Cinnamon, in the Treatment of Breast Cancer”, had used both *in silico* and *in vitro* methodologies for the screening of targets for the bioactive constituents against breast cancer. They had utilized information from various databases, including TCMSP, TCMID, OMIM, etc, and various bioinformatic tools like STRING, Cytoscape, Gene ontology, KEGG, etc for the analysis of relationship between cinnamon phytoconstituents and breast cancer’s targets. Results from both the computational and experimental studies led them to conclude cinnamaldehyde as a promising multifunctional molecule.

Yuan et al., in their article “Transcriptome Profiling and Cytological Assessments for Identifying Regulatory Pathways Associated With Diorcinol N-Induced Autophagy in A3 Cells” had noticed the potential of a fungal originated secondary metabolite, Diorcinol N for the treatment and management of acute lymphoblastic leukemia. The authors had utilized both the bioinformatic tools and *in vitro* methodologies. Diorcinol N was capable of controlling the growth of A3 leukemia cells in a multimodal way.

Wei et al., in their article “Computational and *In Vitro* Analysis of Plumbagin’s Molecular Mechanism for the Treatment of Hepatocellular Carcinoma” had studied Plumbagin, naphthoquinone from *Plumbago zeylanica* L., for the treatment and management of hepatocellular carcinoma. Results from the network pharmacology-based analysis were further validated experimentally using the human hepatocellular carcinoma cell lines, SMMC-7721 and BEL-7404. To combat hepatocellular carcinoma, authors had observed that Plumbagin was able to exert anticancer effects *via* multiple targets and signaling pathways, including that related to oxidative stress, autophagy, and apoptosis.

Jiang et al. In their article “Network Pharmacology and Pharmacological Evaluation Reveals the Mechanism of the Sanguisorba Officinalis in Suppressing Hepatocellular Carcinoma” had identified 41 bioactive ingredients from *Sanguisorba Officinalis* by utilizing some databases like TCMSP and BATMAN-TCM, which then reduced to 12 after filtering through ADME parameters. Network pharmacology approaches were used to analyze those 12 compounds against the selected targets of hepatocellular carcinoma. The results were validated experimentally using human hepatocellular carcinoma cell lines, HepG2, MHCC97H, SMCC7721, and BEL-7404. Based on the bioinformatics analysis, they noticed four compounds like quercetin, kaempferol, mairin, and beta-sitosterol. Overall, they had concluded the significance of *Sanguisorba Officinalis* as an anticancer agent against hepatocellular carcinoma.

Rahman et al., in their review article “Phytochemicals as a Complement to Cancer Chemotherapy: Pharmacological Modulation of the Autophagy-Apoptosis Pathway” had critically analyzed and compiled the knowledge about the plant-based natural products which were having potential to modulate autophagy and apoptosis. The authors had represented the pathways well in the form of interactive figures. They illustrated the detailed pathways for the phytomolecules like 18-α-Glycyrrhetinic acid, Oxyresveratrol, and other polyphenolic and alkaloids. They had also indicated the phytochemicals which have currently been investigated in the clinical trials for the treatment and management of various types of cancer.

Ghanbari-Movahed et al., in their systematic review article “A Systematic Review of the Preventive and Therapeutic Effects of Naringin Against Human Malignancies” had covered several studies indicating the potential of naringin, either alone or synergistic with other drugs or as metal complexes for the treatment and management of various types of human malignancies. They had also specified the plant sources for naringin, all of which belong to the Citrus genus and Rutaceae family. They had reported the significance of naringin against various cancers like bladder, leukemia, lymphoma, brain, breast, cervical, colon, colorectal, esophageal, laryngeal, liver, lung, neuroblastoma, ovarian, prostate, osteosarcoma, chondrosarcoma, melanoma, gastric, and thyroid cancer. Thus, naringin is a multitarget and multiple signaling pathways acting bioactive molecule.

Rasool et al., in their article “Evaluation of the Anticancer Properties of Geranyl Isovalerate, an Active Ingredient of Argyreia nervosa Extract in Colorectal Cancer Cells” had evaluated the effect of geranyl isovalerate against HCT116 and HT29 cell lines related to colorectal cancer. The authors adopted various experimental methodologies for cytotoxicity, live and dead cell detection, JC-1 staining, generation of reactive oxygen species (ROS), genes related to apoptosis, and proteins related to apoptosis. Their results indicated the significance of geranyl isovalerate and generated further interest to assess the translational potential of this WHO-approved food additive as a food supplement.

Sampaio et al., in their systematic review article “Antitumor Effects of Carvacrol and Thymol: A Systematic Review” had covered the literature published from 2003 to 2021 and systematically analyzed the significance of carvacrol, and its isomeric analog, thymol, in the treatment and management of various types of cancer. Authors have recorded the scientific evidence claiming anticancer potential of these two molecules against cancers like that of breast, liver, colon, liver, lung, prostate, etc. Accordingly, authors have indicated various chemopreventive and antimetastatic effects of these molecules as per the recorded literature. Further studies on these molecules can provide vital information about their therapeutic and translational potential to reach the bedside.

Chavda et al., in their review article “Advanced Computational Methodologies Used in the Discovery of New Natural Anticancer Compounds” had discussed the utility aid of computational techniques in the advancement of natural products-based research with a focus on anticancer compounds. In Table 1, they had also discussed 25 natural compounds with details about their anticancer potential, while Table 2 briefly indicated the computational methodologies and tools used during natural anticancer research. Further, the illustrations made by authors represented the global cancer statistics, stepwise structure elucidation, and protocol for fragment-based screening. This article highlighted the crucial role of reliable computational methodologies in the translational process of natural anticancer agents.

Allegra et al., in their article “Evaluation of the IKKβ Binding of Indicaxanthin by Induced-Fit Docking, Binding Pose Metadynamics, and Molecular Dynamics” had assessed the molecular level mechanistic information related to the inhibition of hIKKβ by Indicaxanthin (a betaxanthin from betalain class), using computational tools and techniques like induced-fit docking, binding pose metadynamics, MD simulations, as well as MM-GBSA binding free energy calculation. Previous reports indicated that Indicaxanthin exhibited activity against human melanoma cells by modulating NF-κB and ceasing IκBα degradation. This had generated interest in the team of Mario to explore the mechanisms further on the molecular level, and their results have indeed provided insights and significant knowledge for readers and researchers.

Nuclear factor-κB signalling pathway is a significant and well-known factor influencing immunity, inflammation, cancer, and functioning of the nervous system (Liu et al., 2017; Albensi, 2019). Uddin et al., in their review article “Natural Small Molecules Targeting NF-κB Signaling in Glioblastoma” had discussed the structural and functional properties of NF-κB, role of NF-κB in glioblastoma, as well as the information about small natural products like resveratrol, quercetin, apigenin, isothiocyanates, sulforaphane, etc which were able to target NF-κB in glioblastoma. This article will be interesting for the readers, researchers, and clinicians to explore the translational potential of these natural products for the treatment and management of the most aggressive brain cancer, glioblastoma multiforme.

Crotoxin, a major constituent from the venom of *Crotalus durissus* terrificus, is a known for its pharmacological actions like anticancer activity against breast cancer (Almeida et al., 2021), pain alleviation (Teixeira et al., 2020), antithrombotic (de Andrade et al., 2019), etc, along with its venomous effects. Kato et al., in their research article “Crotoxin Inhibits Endothelial Cell Functions in Two- and Three-dimensional Tumor Microenvironment” studied the antiangiogenic role of the crotoxin in detail and mentioned as to how it will be supportive in the inhibition of tumor progression.

Feng et al. in their research article “Downregulation of ATP1A1 Expression by Panax notoginseng (Burk.) F.H. Chen Saponins: A Potential Mechanism of Antitumor Effects in HepG2 Cells and *In Vivo*”, had evaluated the saponin enriched formulation (commercial source containing notoginsenoside R1, ginsenoside Rg1, ginsenoside Re, ginsenoside Rb1, and ginsenoside Rd) of *Panax notoginseng* (Burk.) F.H. Chen against ATP1A1, an important target for the treatment and management of hepatic carcinoma. They had noticed that the saponin enriched formulation was able to downregulate ATP1A1 and its associated signaling pathway when observed in HepG2 cells. These observations will increase the interest of clinicians’ and researchers’ interest in assessing the translational potential of this saponin enriched formulation of *Panax notoginseng* (Burk.) F.H. Chen.

Singla et al., in their review article “Natural Product-Based Studies for the Management of Castration-Resistant Prostate Cancer: Computational to Clinical Studies”, had covered the literature from last 36 years, and analysed the information about natural products which were effective against castration-resistant prostate cancer (CRPC). They had covered the epidemiology, tumor microenvironment, signaling pathways, genomic targets related information of CRPC. They had covered the computational studies, preclinical studies, and clinical studies that had proved the therapeutic potential of the natural products against CRPC. Clinical studies are undergoing for the phytomolecules like curcumin, EGCG, gossypol, quercetin, lycopene, soy isoflavones, and resveratrol. These observations will increase the interest of clinicians and researchers to assess the translational potential of natural products for the treatment and management of CRPC.

This research topic “Exploration of Natural Product Leads for Multitarget-Based Treatment of Cancer - Computational to Experimental Journey” has been successful in collecting 14 articles: 4 review articles, 2 systematic reviews, and 8 research articles, covering scientific literature pertaining to acute lymphoblastic leukemia, breast cancer, hepatocellular carcinoma, colorectal cancer, melanoma, glioblastoma, and castration-resistant prostate cancer. This research topic has its significance because it has further the knowledge about various natural products like carvacrol, thymol, naringin, diorcinol-N, cinnamaldehyde, plumbagin, crotoxin, geranyl isovalerate, indicaxanthin, *Sanguisorba Officinalis* L., and *Panax notoginseng* (Burk.) F.H. Chen Saponins.

We express our thanks to all the authors for contributing wonderful works in our research topic. To disseminate the knowledge to a wider audience, social media tools are of immense significance. The utilization of hashtags like #INPST, #NPMND and #DHPSP on Twitter or other social media resulted in the greater circulation of the articles and is being considered as digital health promotion (Kletecka-Pulker et al., 2021; Singla, 2021). We strongly encourage the authors and readers to use these hashtags to further promote their scientific literature. Moreover, twitter users can quote @FrontPharmacol to promote their articles published in Frontiers in Pharmacology.
